# Comparison of treatment outcomes between growth-sparing instrumentation and definitive one-stage fusion for EOS patients ages 6–10 years

**DOI:** 10.1007/s43390-023-00731-9

**Published:** 2023-07-22

**Authors:** Charles E. Johnston, David C. Thornberg, Robert Palmer

**Affiliations:** 1 Department of Orthopedics, Scottish Rite for Children, 2222 Welborn Street, Dallas, TX 75219 USA; 2 Pediatric Orthopedics of Southwest Florida, 341 Turnbury Way, Naples, FL 34110 USA

**Keywords:** Early onset scoliosis, Definitive fusion, Growth-sparing treatment, Tweeners

## Abstract

**Purpose:**

To compare objective outcomes for EOS patients age 6–10 years treated by growth-sparing (GS) surgery or definitive one-stage correction and fusion (DF).

**Methods:**

We reviewed surgical, radiographic, PFT’s, and EOSQ-24 outcomes for EOS patients > age 6 at index surgery treated at a single institution, minimum 2-year follow-up. Neuromuscular diagnoses were excluded.

**Results:**

47 patients underwent index surgery between age 6 and 10.9 years. Twenty-one had DF, 26 had GS surgery (13 MCGR, 13 TGR). Diagnoses included 15 congenital, 15 idiopathic, 17 syndromic. Age at index was 9.1 years DF, 7.8 GS (*p* < .001). Follow-up was 63–78 months. 18/26 GS cases converted to DF, 13 due to complications, which occurred in 8/21 DF cases vs 19/26 GS (*p* = .016). DF patients had fewer post-index surgeries (0.6 vs 3.7, *p* < .001). At follow-up there were no differences in curve magnitudes, %correction, T1–12/T1-S1 segment lengths, EOSQ-24 scores or PFTs. 18 patients converting to DF after initial GS had equal outcomes as DF initially. 31 patients > age 8 at index (“tweeners”) were studied separately. 13 had GS surgery (7 MCGR), 18 had DF. At > 60 months follow-up, curve magnitudes, spine lengths, PFT’s, or EOSQ scores were equivalent. DF patients had fewer procedures and complications.

**Conclusion:**

For patients age 6–10.9 years, outcomes were no different at > 5 year follow-up between DF and GS groups. DF patients had fewer total surgeries and complications. Equal outcomes also occurred for tweeners. As a result, GS treatment does not appear to benefit patients > age 8.

## Introduction

Due to widespread recognition that early-onset scoliosis (EOS) is a distinctly different *physiologic* diagnosis from late-onset (e.g., adolescent) scoliosis, it is generally accepted that so-called growth-sparing methods of treatment are indicated for progressive EOS in an attempt to avoid thoracic insufficiency syndrome (TIS) associated with a premature, growth-ending spinal fusion. EOS has been defined as deformity > 10° presenting before age 10 [1], even though most investigators identify the true population at risk for iatrogenic pulmonary impairment as a younger cohort – probably < 6 years old—at the time of fusion. For example, Karol identified such patients being fused at a mean age of 3.3 years, Goldberg at 4.1 years, and Emans and Vitale < age 5 [2–5]. Continuing emphasis on increasing thoracic spine length to achieve a threshold length of 18–20 cm at maturity, depending on diagnosis, has been based on the observation that patients with less than this threshold tended to have poorer pulmonary function tests (PFTs), even though it was equally recognized that multiple factors besides T1–12 length were involved [6, 7]. A recent multi-center study has pursued this length theme, observing higher EOSQ-24 domain scores, including HRQoL domains such as general health, pulmonary function and physical function, as length increases above the thresholds [8].

Simultaneously however, the value of growth-sparing (GS) treatment compared to definitive one-stage correction and fusion (DF) has been questioned in older EOS patients aged 7–10 years [9–14]. In these so-called tweeners, where spine length—actual and potential—may be sufficient to ensure adequate thoracic volume and respiratory function, especially when deformity is effectively corrected [7], the gain of an additional 1–2 cm of thoracic length via a GS method may not provide objective improvement in either deformity management, pulmonary function or patient-reported outcome measures compared to a definitive one-stage correction and fusion. This may be especially obvious when complications and unplanned returns to the operating room (UPROR) associated with GS methods are known to be more frequent.

The purpose of this study was to compare the outcomes of distraction-based growth-sparing surgical methods and definitive one-stage correction and fusion in patients who were at least 6 years old at the index procedure. In addition, due to the evolving management of EOS in “tweeners,” we studied a narrower, older cohort of patients > 8 years of age, hypothesizing that there would be no differences in radiographic parameters, PFTs or patient-reported outcomes between the GS and DF patients once they had reached these ages at the time of the index procedure.

## Materials and methods

### Study design

We performed a longitudinal study of patients from a single institution with EOS undergoing surgery.

### Level of evidence

Level II—Prognostic.

### Study patients

We identified patients aged 6.0–10.9 years at the time of their index surgical procedure performed at a single institution between 2011 and 2020, with minimum 2-years follow-up from the index procedure. Patients with neuromuscular etiologies were excluded due to inability to perform PFT’s of reliable validity. Outcomes measures included preoperative and last follow-up radiographic measurement of major coronal and sagittal curve magnitude, T1–12 height, T1-S1 length; the number of vertebral segments spanned by the DF and GS constructs; most recent pulmonary function test (PFT); and most recent scores on the 24-item Early Onset Scoliosis Questionnaire (EOSQ-24). Pre-treatment PFTs in study patients were sporadically obtained in the early years of the study period, and the EOSQ-24 was not available prior to 2018, limiting pre-treatment score availability. We characterized all surgeries after index, documented complications, and unexpected return to the operating room (UPROR).

### Statistical analysis

Statistical analyses were completed in SPSS Statistics (version-24; IBM). Analysis of continuous variables, such as Cobb angle, age at surgery, T1–T12 and T1-S1, were first examined for normality with a Shapiro–Wilk-Test, and a *t* test and Mann–Whitney-*U* test were used for 2-group comparisons as appropriate.

## Results

47 patients (8 male, 39 female) fulfilled inclusion criteria: 21 underwent DF as the index procedure, while 26 underwent GS, which included 13 magnetic-controlled growing rods (MCGR) and 13 “traditional” growing rods (TGR). Diagnoses included 15 subjects with congenital scoliosis (DF 8, GS 7), 15 with idiopathic EOS (DF 5, GS 10), and 17 with syndromic etiologies (DF 9, GS 8).

For the cohort > age 8 at surgery, there were 31 patients (26 females), with 10 congenital, 11 idiopathic, and 10 syndromic diagnoses. 18 subjects underwent index DF while 13 had a GS procedure (7 MCGR, 6 TGR).

In the full cohort, patients in the DF group were older—9.1 years vs. 7.8 in the GS group, *p* < 0.001 (Table 1). Preoperative T1-S1 length was also greater in the DF group (29.2 vs 26.7 cm, *p* = 0.045). There were no other differences between the two groups preoperatively, including patient height, weight, BMI, coronal and sagittal plane magnitudes (Cobb), and thoracic spine length. Mean follow-up was 63 months in the DF group and 78 months in the GS group, with no difference in age at most recent follow-up. Of the 16 patients < age 8 at surgery, 13 had GS procedures while only 3 had DF. Of the 26 GS patients, 18 have subsequently undergone DF, 15 of which were performed because of complications of GS methods. At follow-up, 14 of 47 patients were still considered immature, Risser ≤ 2 and/or open tri-radiate cartilage.

**Table 1:** Radiographic and Patient Data (Total Cohort)

	Parameter	Index surgical procedure				*p*
		Definitive fusion		Growth sparing		
		*N*	Mean (range)	*N*	Mean (range)	
Preoperative	Major curve cobb	21	73 (47–105)	26	81 (47–137)	0.4
	T1-12 height (mm)	21	166 (126–215)	26	155 (83–201)	0.2
	T1-S1 length (mm)	21	292 (221–354)	26	267 (186–341)	0.045
	Max kyphosis	20	48 (5–81)	26	43 (− 19 to 114)	0.5
	Age at surgery (years)	21	9.1 (6.1–10.9)	26	7.8 (6–10)	< 0.001
	Curve Levels	21	12 (5–15)	26	13.7 (10–19)	0.1
Radiographic follow-up	Age (years)	21	14.5 (11.1–17.8)	26	14.3 (11–22)	0.4
	Time since surgery (months)	21	63.4 (26–101)	26	78 (36–189)	0.2
	Major curve cobb	21	32 (4–76)	26	34 (13–60)	0.7
	T1-12 height (mm)	21	199 (156–241)	26	204 (117–262)	0.6
	T1-S1 length (mm)	21	340 (271–446)	26	346 (247–428)	0.6
	Max Kyphosis	21	37 (9–55)	26	33 (− 2 to 58)	0.5
	Instrumented levels	21	12.8 (5–18)	26	14.3 (11–19)	0.1
	Additional surgeries	21	0.6 (0–4)	26	3.7 (0–12)	< 0.001
PFT follow-up	Age (years)	21	13.6 (10.7–17.8)	26	12.8 (9.3–18.2)	0.2
	Time since surgery (months)	21	53 (27–85)	26	60 (25–144)	0.6
	FVC Actual	21	1.9 (0.5–6)	26	1.6 (0.3–3.4)	0.3
	FVC percent predicted	21	58 (15–158)	26	51 (14–101)	0.3
	FEV1 actual	21	1.4 (0.5–2.3)	26	1.4 (0.3–3.1)	0.6
	FEV1 percent predicted	21	51 (16–77)	26	50 (14–111)	0.9
Changes at follow-up	% Correction major curve cobb		54 (− 29 to 94)		56 (− 13 to 82)	0.9
	Change in T1–12 height (mm)		33 (− 6 to 76)		50 (10–101)	0.021
	Change in T1-S1 length (mm)		48 (11–118)		79 (43–146)	< 0.001
	Change in levels		1 (0–7)		0.8 (− 2 to 7)	0.8
	ΔFVC actual		0.7 (0.05–1.7)		0.7 (0.07–1.9)	0.8
	ΔFVC % predicted		− 3.9 (− 63 to 54)		− 10 (− 87 to 27)	0.9
	ΔFEV1 actual		0.5 (0.09–1.2)		0.6 (0.07–1.8)	0.8
	ΔFEV1% predicted		− 2.6 (− 24 to 39)		− 7.5 (− 40 to 36)	0.5

At the most recent follow-up for the full cohort, there were no differences in % correction, coronal or sagittal curve magnitudes between the two groups (Table 1). T1–12 and T1-S1 at follow-up were similar, by virtue of a larger percentage amount of length gained in both spine segments by the GS procedures compared to DF (*p* < 0.001 for T1-S1). Although the number of segments within the main curve preoperatively, and the number of segments spanned by the instrumentation at follow-up was slightly greater in the GS cohort, this was not statistically significant (Table 1). Additionally there was no difference in segments spanned at follow-up in the GS patients who underwent conversion compared to patients who had DF initially (Table 3). DF patients averaged 0.6 procedures (range 0–4) procedures following index surgery, significantly fewer than the GS patients who averaged 3.7 (range 0–12, *p* < 0.001). 8/21 (38%) DF patients suffered complications during treatment, compared to 19/26 (73%, *p* = 0.016) in the GS group. Three DF patients required unplanned surgery for adding-on, while 13 GS patients had complications which led to conversion to DF (Table 4). Thirteen of 21 DF patients had no other surgery beside the index procedure. Eight of the 26 GS patients have retained their initial implants at follow-up.

For the 31 patients > age 8 at surgery, the DF group was again older—9.5 years vs 8.6 for GS, *p* = 0.01—and had longer T1-S1 length (29.5 vs 27.5 cm, *p* = 0.003, Table 2). There were no other preoperative differences in patient or radiographic parameters (Cobb angle, spine lengths). 7 of 13 GS subjects were treated with MCGR. At follow-up, averaging 61 months for the DF patients and 63 for GS, the only differences between groups were increased kyphosis in the DF group and greater incremental gains in both T1-12 and T1-S1 segment lengths in GS (Table 2). There was no difference between DF and GS groups in the number of segments in the preoperative curve or spanned by instrumentation at follow-up. The DF group again had a significantly fewer number of procedures—0.6 vs 3.2 (*p* < 0.001). As was the case for the full cohort, 33% of DF patients had complications, significantly less than 77% for GS (*p* = 0.01). Three of 5 DF complications were progressive deformity due to distal adding-on, similar to crankshaft phenomenon, requiring extending the instrumentation distally. Progressive deformity within the instrumented segments in the other 15 patients was not observed, in part due to adding anterior fusion to the posterior instrumented fusion in 8 of the 15 patients.

**Table 2:** Radiographic and Patient Data (8.0 – 10.9 Years Old Cohort)

	Parameter	Index surgical procedure				*p*
		Definitive fusion		Growth sparing		
		*N*	Mean (range)	*N*	Mean (range)	
Preoperative	Major curve cobb	18	75 (47–105)	13	82 (47–137)	0.2
	T1-12 height (mm)	18	167 (135–215)	13	161 (83–201)	0.5
	T1-S1 length (mm)	18	295 (233–354)	13	275 (186–341)	0.003
	Max kyphosis	18	47 (5–75)	13	42 (14–114)	0.2
	Age at surgery (years)	18	9.5 (8.1–10.9)	13	8.6 (8.1–10)	0.01
	Curve levels	18	12.6 (5–15)	13	13.7 (11–19)	0.4
Radiographic follow-up	Age (years)	18	14.6 (11.1–17.8)	13	13.9 (11.2–18.5)	0.8
	Time since surgery (months)	18	61 (26–101)	13	63 (36–123)	0.8
	Major curve cobb	18	29 (4–50)	13	28 (13–52)	0.4
	T1-12 height (mm)	18	201 (162–241)	13	209 (156–247)	0.1
	T1-S1 length (mm)	18	341 (271–446)	13	352 (297–397)	0.4
	Max kyphosis	18	37 (18–55)	13	29 (7–50)	0.005
	Instrumented levels	18	13.1 (5–18)	13	14.1 (11–19)	0.4
	Additional surgeries	18	0.6 (0–4)	13	3.2 (0–8)	< .001
PFT follow-up	Age (years)	18	13.8 (11.1–17.8)	13	13.2 (10.9–15.8)	0.5
	Time since surgery (months)	18	51 (26.7–84.9)	13	55 (30.1–90.1)	0.011
	FVC actual	18	1.8 (0.8–2.6)	13	1.7 (0.3–3.4)	0.4
	FVC percent predicted	18	56 (27–79)	13	51 (14–101)	0.8
	FEV1 actual	18	1.5 (0.7–2.3)	13	1.5 (0.3–3.1)	0.3
	FEV1 percent predicted	18	54 (30–77)	13	52 (14–111)	0.2
Changes at follow-up	% Correction major curve cobb		60 (25–94)		64 (32–80)	0.008
	Change in T1–12 height (mm)		34 (− 6 to 76)		48 (20–101)	0.2
	Change in T1-S1 length (mm)		46 (11–118)		77 (43–146)	0.004
	Change in levels		0.7 (0–5)		0.6 (0–4)	0.9
	ΔFVC actual		0.68 (0.05–1.73)		0.78 (0.07–1.92)	0.8
	ΔFVC % predicted		− 3.91 (− 63 to 54)		-3.4 (-32–27)	0.9
	ΔFEV1 actual		0.55 (0.09–1.22)		0.63 (0.07–1.81)	0.8
	ΔFEV1% predicted		− 2.64 (− 24 to 39)		− 5 (− 40 to 36)	0.5

We then compared the outcomes of the 21 patients initially treated by DF to the 18 patients who had GS treatment but were converted to DF. 11/18 were treated with MCGR while 7 had TGR. At follow-up (63 months for DF, 76 months for conversion DFs), there were no differences in any radiographic measures, and no difference in number of instrumented segments between DF and conversion groups (Table 3). Not surprisingly, DF patients had a 38% complication rate (8/21), compared to 83% (15/18) conversion patients (*p* = 0.008, Table 4). Of the 15 patients with complications, 8 patients with MCGRs and 5 patients with TGRs underwent conversion due to a complication.

**Table 3:** Radiographic and Patient Data (GS Conversion to Fused at Follow-Up)

Parameter		Index surgical procedure				*p*
		Definitive fusion		Growth sparing		
		*N*	Mean (range)	*N*	Mean (range)	
Preoperative	Major curve cobb	21	73 (47–105)	18	77 (47–131)	0.6
	T1-12 height (mm)	21	166 (126–215)	18	164 (114–201)	0.8
	T1-S1 length (mm)	21	292 (221–354)	18	279 (205–341)	0.3
	Max kyphosis	20	48 (5–81)	18	43 (15–75)	0.4
	Age at surgery (years)	21	9.1 (6.1–10.9)	18	7.9 (6.3–10)	0.003
	Curve levels	21	12 (5–15)	18	13.4 (10–17)	0.2
Radiographic follow-up	Age (years)	21	14.5 (11.1–17.8)	18	14.3 (11–19.6)	0.8
	Time since surgery (months)	21	63 (26–101)	18	76 (40–138)	0.2
	Major curve cobb	21	32 (4–76)	18	32 (13–60)	0.9
	T1-12 height (mm)	21	199 (156–241)	18	211 (156–262)	0.2
	T1-S1 length (mm)	21	340 (271–446)	18	354 (289–428)	0.3
	Max kyphosis	21	37 (9–55)	18	36 (7–58)	1.0
	Instrumented levels	21	12.8 (5–18)	18	14.2 (11–16)	0.1
	Additional surgeries	21	0.6 (0–4)	18	3.3 (1–11)	< 0.001
PFT follow-up	Age (years)	21	13.6 (10.7–17.8)	18	12.6 (9.5–15.8)	0.1
	Time since surgery (months)	21	53 (26.7–84.9)	18	56 (30–106)	0.7
	FVC actual	21	1.9 (0.5–6)	18	1.7 (0.3–3.4)	0.4
	FVC percent predicted	21	58 (15–158)	18	50 (14–74)	0.3
	FEV1 actual	21	1.4 (0.5–2.3)	18	1.4 (0.3–2.3)	0.6
	FEV1 percent predicted	21	51 (16–77)	18	48 (14–70)	0.5
Changes at follow-up	% Correction major curve cobb		54 (− 29 to 94)		55 (-13–82)	0.9
	Change in T1-12 height (mm)		33 (− 6 to 76)		47 (18–100)	0.1
	Change in T1-S1 length (mm)		48 (11–118)		74 (43–125)	0.002
	Change in levels		1 (0–7)		1 (− 2 to 7)	0.9
	ΔFVC actual		0.7 (0.1–1.7)		0.7 (0.6–0.9)	0.7
	ΔFVC % predicted		− 3.9 (− 63–54)		− 7.8 (− 32 to 11)	0.7
	ΔFEV1 actual		0.5 (0.1–1.2)		0.5 (0.3–0.7)	1.0
	ΔFEV1% predicted		− 2.6 (− 24–39)		− 12.1 (− 40 to 4)	0.2

**Table 4:** Complication and Patient Data

		Cohort								
		Total			8.0–10.9 Years Old			Fused at final follow-up		
		Definitive fusion	Growth sparing	*p*	definitive fusion	Growth sparing	*p*	Definitive fusion	Growth sparing	*p*
		*N*	*N*		*N*	*N*		*N*	*N*	
EOS classification	Congenital	8	7	0.5	7	3	0.2	8	5	0.6
	Idiopathic	5	10		4	7		5	7	
	Syndromic	8	9		7	3		8	6	
Complication	No	13	7	0.016	13	3	0.011	13	3	0.008
	Yes	8	19		5	10		8	15	
					Complication led to Fusion		No		2	–
							Yes		13	
					Complication led to fusion		Instrumentation		10	–
							Medical		2	
							Progression		1	

### EOSQ-24 scores (Table 5)

**Table 5:** EOSQ Data

Domain		Cohort								
		Total			8.0–10.9 years old			Fused at final follow-up		
		Definitive fusion	Growth sparing	*p*	Definitive fusion	Growth sparing	*p*	Definitive fusion	Growth sparing	*p*
		Mean (range)	Mean (range)		Mean (range)	Mean (range)		Mean (range)	Mean (range)	
Preoperative	General health	72 (50–88)	75.3 (38–100)	0.7	69.7 (50–88)	72.8 (62.5–88)	0.8	72 (50–88)	77.7 (62.5–100)	0.6
	Pain discomfort	73.5 (50–100)	77.2 (50–100)	0.6	73.3 (50–100)	75.2 (63–100)	0.3	73.5 (50–100)	81.3 (50–100)	0.3
	Pulmonary function	89.1 (50–100)	81.4 (50–100)	0.2	89.3 (50–100)	80.1 (63–100)	0.2	89.1 (50–100)	81.4 (50–100)	0.2
	Transfer	87.5 (50–100)	75 (25–100)	0.2	85.7 (50–100)	70 (25–100)	0.2	87.5 (50–100)	72.5 (25–100)	0.2
	Physical discomfort	84.4 (17–100)	86.8 (33–100)	0.8	82.2 (17–100)	93.4 (75–100)	0.7	84.4 (17–100)	85.8 (33–100)	0.9
	Daily living	78.3 (25–100)	79.4 (38–100)	0.9	76.9 (25–100)	77.7 (50–100)	0.6	78.3 (25–100)	76.5 (38–100)	0.9
	Fatigue energy level	86 (38–100)	72 (13–100)	0.2	84 (38–100)	70.1 (38–100)	0.4	86 (38–100)	72.7 (13–100)	0.3
	Emotion	75.1 (25–100)	67.8 (25–88)	0.6	71.5 (25–100)	62.6 (25–75)	0.8	75.1 (25–100)	66.4 (25–88)	0.5
	Parental impact	79.4 (30–100)	70 (35–95)	0.2	76.4 (30–95)	82 (50–95)	0.7	79.4 (30–100)	70 (35–95)	0.2
	Financial impact	71.9 (0–100)	56.4 (0–100)	0.3	67.9 (0–100)	70.4 (2–100)	0.9	71.9 (0–100)	52.7 (0–100)	0.2
	Satisfaction	79.8 (25–100)	67.8 (50–100)	0.2	76.9 (25–100)	75.1 (62.5–100)	0.6	79.8 (25–100)	66.4 (50–100)	0.2
Follow-up	Time since surgery (months)	52.7 (26–101)	63.7 (30–103)	0.2	51.2 (26–101)	59.7 (30–103)	1.0	52.7 (26–101)	62.6 (30–102)	0.3
	General health	70.5 (37.5–100)	81.3 (50–100)	0.1	73.8 (50–100)	81.9 (62.5–100)	0.3	70.5 (37.5–100)	82.2 (62.5–100)	0.1
	Pain discomfort	69.3 (25–100)	71.9 (12.5–100)	0.7	73.8 (37.5–100)	73.9 (12.5–100)	0.8	69.3 (25–100)	75.9 (25–100)	0.5
	Pulmonary function	80.7 (12.5–100)	76.9 (25–100)	0.4	87.5 (75–100)	89.8 (25–100)	0.1	80.7 (12.5–100)	70.6 (25–100)	0.8
	Transfer	90.9 (25–100)	83.8 (50–100)	0.3	90 (25–100)	93.2 (50–100)	0.9	90.9 (25–100)	82.1 (50–100)	0.2
	Physical discomfort	81.1 (25–100)	78.3 (0–100)	0.9	84.2 (25–100)	87.1 (0–100)	0.7	81.1 (25–100)	81 (41.7–100)	1.0
	Daily living	81.8 (25–100)	79.4 (37.5–100)	0.9	81.3 (25–100)	86.4 (37.5–100)	0.6	81.8 (25–100)	84.8 (37.5–100)	0.7
	Fatigue energy level	69.3 (25–100)	77.5 (37.5–100)	0.3	73.8 (37.5–100)	83 (50–100)	0.2	69.3 (25–100)	76.8 (37.5–100)	0.4
	Emotion	77.3 (25–100)	80.1 (25–100)	0.9	81.3 (25–100)	84.1 (25–100)	0.8	77.3 (25–100)	78.6 (25–100)	1.0
	Parental impact	75.3 (8–100)	80 (35–100)	1.0	77.3 (8–100)	86.4 (50–100)	0.7	75.3 (8–100)	79.3 (35–100)	0.9
	Financial impact	93.2 (50–100)	78.8 (25–100)	0.2	92.5 (50–100)	90.9 (25–100)	1.0	93.2 (50–100)	76.8 (25–100)	0.2
	Satisfaction	79.5 (37.5–100)	71.3 (25–100)	0.4	82.5 (37.5–100)	79.5 (50–100)	0.7	79.5 (37.5–100)	71.4 (25–100)	0.4

Preoperative EOSQ-24 scores showed no differences in any domains between all the groups. However, as noted earlier, this instrument was not available to be administered prior to 2018. Consequently, data was only available for 20 patients.

There were no significant differences between EOSQ-24 scores at follow-up in the entire cohort. Selected specific domain mean scores included: general health—DF 71 GS 81; pulmonary function—DF 81 GS 77; physical discomfort—DF 81 GS 78; fatigue/energy: DF 69 GS 77; emotion—DF 77 GS 80; parental impact—DF 75 GS 80; and satisfaction—DF 80 GS 71. Comparison of the follow-up scores in the surgery > 8 years group and the comparative scores between the initial DF and the conversion DF groups also showed no differences in any of the domains.

### Pulmonary function tests

There were also no differences in absolute PFT volumes (liters) at follow-up—FVC: DF 1.9 (0.5–6), GS 1.6 (0.3– 3.4); FEV1: DF 1.4 (0.5–2.3), GS 1.4 (0.3–3.1). Similarly, there were no differences in % predicted volumes—% FVC: DF 58 (15–77), GS 51 (14–74); %FEV1: DF 51 (16–77), GS 50 (14–70), Table 1. In the > age 8 cohort, the absolute and % predicted volumes were similar to the full cohort and showed no differences (Table 2). Finally, no differences in PFT parameters were found between the patients treated by DF and those converted after initial GS treatment (Table 3).

## Discussion

Growth-sparing surgical management for EOS patients has become widely accepted over the 20 years since reports that early growth-ending spinal fusion could not only result in respiratory impairment leading to thoracic insufficiency syndrome, but also was typically not definitive management [2, 3]. Historical categorization of idiopathic deformity into early onset (0–5 years) and later onset (> age 5) recognized the essential difference between these entities based on this age threshold [15] and the ominous respiratory consequences of untreated early-onset deformity [18, 19]. Additional studies [4, 5] confirmed that the < 5 age group were the most susceptible to respiratory impairment associated with early fusion. The SRS Growing Spine Committee recognized the essential differences in treatment strategies and outcomes characteristic of the < age 5 patient group, but nevertheless defined EOS as deformity presenting < age 10, because “treatment principles” for the 6–10 years old group more “closely resembled” those accepted for the < 5 age group [1], implying that growth-sparing methods consequently were always appropriate.

Recently a reappraisal of these principles for the older EOS patient is underway [6, 9–14], primarily observing that repeated efforts to gain an extra 1–2 cm of thoracic length by GS procedures failed to provide measurable benefit in terms of curve correction and better patient reported outcomes, and at the cost of significantly greater number of surgical procedures, complications and UPRORs. Since the age ranges in these studies for index definitive fusion coalesced around age 10 [9, 11–14], the lack of benefit for GS procedures at this upper EOS age range suggests that the < age 10 limit may need to be revised downward. A search of current literature found that the lowest published ages for index DF go as young as 5.1 [7] and 7.3 [10] years.

The current study provides further evidence to support the direction of treatment away from GS methods in the older EOS population. At mean follow-up of 63 and 78 months (minimum 24) since index surgery for DF and GS patients, respectively, PFTs and EOSQ-24 scores were equivalent, as were major curve magnitudes and thoracic and total spine lengths. The only differences detected were a greater incremental length change in the GS cohort and significantly fewer procedures and complications in the DF cohort (Tables 1, 4). Furthermore, by focusing more on patients > 8 years at initial surgery, outcomes equivalence between DF and GS cohorts at 61 and 68 months follow-up is again demonstrated, with the only differences being the same increased incremental change in T1-S1 length and the significantly fewer procedures and complications in the DF group (Tables 2, 4). Finally, the end results of conversion to definitive fusion for 18 subjects originally treated by GS technique, when compared to those treated by DF initially, showed no differences in curve magnitudes, spine segment lengths, or segments spanned by instrumentation, and the functional outcomes (PFTs, EOSQs) at follow-up (Tables 3, 5).

Frequently termed “tweeners,” the > 8 age group constitutes a distinct cohort best defined by chronological age (8–10 years in females, 9–11 years in males), Sanders score [17] less than 4, open triradiate cartilage, and bone age (8 + 10–10 + 10 for females, < 12 for males) [16]. These patients may now have compelling indications for index definitive fusion, as the age and maturity criteria at which fusion no longer creates concern for respiratory impairment leading to TIS, based on a perceived excessive shortening of the thoracic spine, have now been clarified. Furthermore, as the length of instrumentation (number of spine segments spanned) was essentially the same for the two treatment groups, and conversion definitive fusion (Table 3) did not require further extension of the implants, there was no advantage to the GS method. On the contrary, since GS treatment predictably incurs multiple surgeries, as observed in this study, related to planned or unplanned procedures and higher complication rates, a single definitive correction and fusion would be superior if the outcome compared to GS surgery is equivalent. The results of this study confirm that equivalence and suggest that future use of GS methods should be minimized in the 8–10 year age group (“tweeners”) and reserved primarily for patients younger than 8 years. The results of this study suggest that future use of GS methods be more carefully considered since the attempt to gain additional length did not provide additional benefit. Furthermore, except for the 3 DF patients in the tweener group who had junctional adding-on after posterior fusion only, requiring extending the instrumentation distally, progressive deformity due to crankshaft in the DF patients was not observed. Curve control provided by a single DF in this cohort effectively allays concerns of ineffective curve management observed in the studies of Karol et al. and Goldberg et al. [2, 3].

Of additional concern considering the equivalence of the PFT outcomes in the two cohorts is the rather modest outcomes of 50–55% predicted volumes reported in this study (Tables 1, 2, 3). Continued deterioration of pulmonary function in young adults fused early (mean age 3.3 years) has been reported by Bouton et al. [20], who documented an interval decline from 55% predicted FVC and FEV1—approximately the same mean %predicted values found in this study, regardless of treatment group—to 42% predicted volumes at an additional 12 years follow-up for patients originally in the study of Karol et al. [2]. That 50–55% predicted PFT outcome in the tweener population is all that is achieved by either single definitive fusion or a growth-sparing technique, which provide seemingly satisfactory Cobb correction (< 30°) and T1-12 length (20 cm, Table 2), is a fairly ominous result at this stage of adolescence, in view of Bouton’s report. It appears that current treatment methods lack efficacy if such PFT values become the norm for EOS management results.

The main limitations of this study, as a single-institution effort, are a relatively small number of patients, and likely treatment bias, inherent to a retrospective study, based on patient age concerns. In the full cohort, DF patients were significantly older, by an average of 1.3 years, than those selected for GS methods. Only three patients < age 8 out of 16 were selected for an DF, compared to 18 of 31 over age 8, an indication of preference for DF in the older age range of traditionally-defined EOS. Curve magnitudes and characteristics were similar with the exception that the younger patients in the GS group also had shorter T1-S1 lengths. By specifically re-analyzing the cohort of patients > 8 years at index—a cohort approximating the tweener population—we were closer to age parity between the two groups—DF mean age 9.5 years, GS 8.6—but still with some bias toward younger aged subjects for GS technique. Also, 30% of the patients (14/47) are still considered immature (tri-radiate cartilage open, Risser ≤ 2) at last follow-up, indicating that additional future surgeries remain a distinct possibility. Nevertheless, in spite of these limitations, the equivalent objective outcomes in pulmonary function tests, patient-reported outcomes, and radiographic curve and length parameters at a mean follow-up of at least 5 years suggest that growth-sparing treatment be limited in tweener cases to those where additional spine length is subjectively desirable even though functional benefit is not demonstrable, and the documented risks of multiple procedures, complications and unplanned returns to surgery are clearly understood.

## Data Availability

Data will not be available.
